# Genome-Wide Characterization of the PEBP Gene Family in Chinese Plum (*Prunus salicina* Lindl.) and Expression Profiling During Floral Bud Development and Flowering

**DOI:** 10.3390/biology15191786

**Published:** 2026-10-08

**Authors:** Chen Lu, Qianju Wang, Fei Han, Shouyi Chen, Zhijun Peng

**Affiliations:** 1 Guizhou Institute of Pomology Science, Guiyang 550025, China; fruitlu96@126.com (C.L.);; 2 Guizhou Key Laboratory of Molecular Breeding for Characteristic Horticultural Crops, Guiyang 550025, China

**Keywords:** *Prunus salicina*, PEBP gene family, genome-wide identification, floral bud development, gene expression, subcellular localization

## Abstract

The phosphatidylethanolamine-binding protein (PEBP) family plays a crucial role in regulating flowering and other developmental processes, yet it remains insufficiently characterized in Chinese plum (*Prunus salicina* Lindl.). A comprehensive genome-wide analysis identified seven candidate *PsPEBP* genes, which are categorized into the TFL1-like, FT-like, MFT-like, and AT5G01300-related groups. These genes are distributed across five chromosomes and encode proteins ranging from 100 to 174 amino acids in length. Analyses of sequence, motif, gene structure, and synteny revealed conserved characteristics, although the abbreviated PsPEBP4 model requires further validation. The nonsynonymous-to-synonymous substitution rate ratios, ranging from approximately 0.03 to 0.35, suggest the influence of purifying selection. The upstream regions of these genes contain putative regulatory elements associated with light, hormones, development, and stress responses. Transcriptome analysis during fruit development demonstrated distinct temporal expression patterns among the *PsPEBP* genes. Quantitative reverse transcription PCR analysis demonstrated distinct expression patterns throughout the stages of floral bud development and flower opening (F1–F5). *PsPEBP1*, *PsPEBP2*, and *PsPEBP7* exhibited their highest relative expression levels at stage F1, followed by a subsequent decline. In contrast, *PsPEBP3* and *PsPEBP6* reached their expression peak at stage F2, while *PsPEBP5* peaked at stage F3. Notably, *PsPEBP4* expression decreased at stage F2 and then progressively increased from stages F2 to F5. Subcellular localization studies revealed that PsPEBP2 is present in both the cytoplasm and nucleus of *Nicotiana benthamiana* leaf cells. These findings lay the groundwork for further investigation into the roles of *PsPEBP* genes in reproductive development.

## Simple Summary

Floral transition and floral organ development influence yield and fruit quality in Chinese plum (*Prunus salicina*), but the roles of the phosphatidylethanolamine-binding protein (PEBP) family in these processes remain unclear. Seven candidate *PsPEBP* genes were identified, and their evolutionary relationships, structural features, and potential regulatory elements were characterized. Expression analyses revealed distinct patterns during fruit development, floral bud development, and flower opening, suggesting potential roles in reproductive development. Subcellular localization analysis indicated that the PsPEBP2 protein was localized to both the cytoplasm and nucleus in tobacco leaf cells. These findings provide a foundation for investigating the functions of *PsPEBP* genes in flowering regulation in Chinese plum.

## 1. Introduction

The shift from vegetative to reproductive growth is contingent upon the integration of environmental cues and intrinsic developmental programs. In *Arabidopsis thaliana*, genetic analyses of the *CONSTANS* and *FLOWERING LOCUS C* genes have elucidated the convergence of photoperiodic and vernalization pathways in the regulation of flowering [1,2]. The phosphatidylethanolamine-binding protein (PEBP) family encompasses FLOWERING LOCUS T (FT), a pivotal promoter of flowering, and TERMINAL FLOWER 1 (TFL1), which acts to delay flowering and preserve inflorescence meristem identity [3,4]. These opposing functions render PEBP proteins critical determinants of reproductive timing and plant architecture. Plant PEBPs have historically been categorized into three primary groups: FT-like, TFL1-like, and MOTHER OF FT AND TFL1 (MFT)-like, as noted by previous studies [5]. The six traditionally characterized members within *Arabidopsis thaliana* include FLOWERING LOCUS T (FT), TWIN SISTER OF FT (TSF), TERMINAL FLOWER 1 (TFL1), BROTHER OF FT AND TFL1 (BFT), ARABIDOPSIS THALIANA CENTRORADIALIS (ATC), and MFT. TSF exhibits partial functional redundancy with FT, while BFT and ATC are implicated in the suppression of flowering and the regulation of meristem development [6,7,8]. MFT is involved in seed germination through its role in abscisic acid signaling pathways [9]. In 2024, the gene *AT5G01300* was designated as *STEPMOTHER OF FT AND TFL1* (*SMFT*) and was classified into a newly identified fourth PEBP lineage. *Arabidopsis* mutants for this gene displayed modified seed dormancy and germination responses, yet did not exhibit any discernible changes in flowering time [10]. Consequently, this study delineates four distinct groups: FT-like, TFL1-like, MFT-like, and AT5G01300-related, while acknowledging that these lineages may not share equivalent developmental functions.

The molecular functions of FT and TFL1 exemplify how closely related PEBPs can lead to distinct developmental outcomes. In *Arabidopsis*, the FT protein translocates from the leaves to the shoot apex, and its rice homolog, Hd3a, similarly functions as a mobile flowering signal [11,12]. At the *Arabidopsis* shoot apex, FT interacts with the bZIP transcription factor FD to promote the expression of genes associated with floral meristem identity [13]. In rice, Hd3a, in conjunction with 14-3-3 proteins and OsFD1, forms a florigen activation complex [14]. Within the *Arabidopsis* shoot meristem, TFL1 competes with FT for regulatory activity associated with FD [15,16]. Experiments involving amino acid substitution and protein-domain exchange have further elucidated that specific residues and an external loop are critical to the contrasting activities of FT and TFL1 [17,18]. Consequently, sequence similarity alone is inadequate to determine the function of an uncharacterized PEBP protein. PEBP proteins have both conserved and diversified functions across crop species and developmental contexts. In tomatoes, the *SELF-PRUNING* gene, an ortholog of *TFL1*, plays a crucial role in regulating shoot determinacy, whereas the *FT* ortholog, *SINGLE FLOWER TRUSS*, is responsible for generating systemic flowering signals [19,20]. Heterozygosity for loss-of-function alleles of *SINGLE FLOWER TRUSS* has been shown to enhance yield in the tomato genotypes studied [21]. Additionally, the functional divergence among *FT* paralogs has been a significant factor in the evolution of flowering time during the domestication of sunflowers [22]. It is noteworthy that classification as FT-like does not uniformly indicate floral promotion; for instance, in sugar beet, *BvFT1* acts as a repressor of flowering, while *BvFT2* serves as a promoter [23]. In poplar, the *CO*/*FT* module integrates photoperiod with seasonal growth, with *FT1* and *FT2* assuming distinct roles in reproductive initiation and vegetative growth [24,25]. These instances underscore the critical importance of considering lineage, tissue type, and developmental stage when interpreting PEBP expression patterns.

Within the Rosaceae, a comparative study of nine tree species documented variation in PEBP copy number, duplication patterns, and expression profiles, and inferred an FT-associated coexpression module linked to floral bud break and blooming in *Prunus mume* [26]. This module represents an expression-based hypothesis rather than an experimentally established regulatory mechanism. Functional evidence is available in apple: *MdFT1* overexpression induces precocious flowering, whereas antisense suppression of *MdTFL1* reduces the juvenile phase [27,28]. Expression of poplar *FT1* in European plum (*Prunus domestica*) also alters flowering, tree architecture, and dormancy behavior [29]. These findings provide a comparative framework for studying *PEBP* genes in Chinese plum, while leaving their individual contributions in this species to be resolved. The Chinese plum (*Prunus salicina* Lindl.) is a diploid fruit tree, and its reproductive development is crucial for fruit production. Controlled pollination experiments conducted on the ‘Fengtangli’ cultivar have demonstrated that the source of pollen significantly affects both fruit set and fruit characteristics [30]. Chromosome-level genome assemblies serve as valuable resources for examining developmental gene families. Furthermore, studies on the low-chill cultivar ‘Sanyueli’ have elucidated the molecular processes linked to the chilling requirement of flower buds [31,32]. Consequently, characterizing the sequence diversity of PEBP genes, along with their developmental expression, can facilitate the identification of candidate genes for investigating reproductive development in the Chinese plum.

In this study, seven candidate *PsPEBP* genes were identified and characterized through comprehensive analyses, including phylogenetic assessment, gene structure examination, conserved motif identification, upstream regulatory element analysis, synteny evaluation, and sequence divergence investigation. Fruit expression patterns were analyzed using publicly available transcriptome data from ‘Furongli’ [33]. Additionally, an independent quantitative reverse transcription PCR (qRT-PCR) analysis was conducted in ‘Fengtangli’ to examine five distinct stages of floral bud development and flower opening: F1 (early floral bud), F2 (swollen floral bud), F3 (elongated closed floral bud), F4 (pre-anthesis with visible petals), and F5 (open flower). These stages represent the developmental progression following floral bud formation. Furthermore, the subcellular localization of PsPEBP2 was investigated through transient expression in *Nicotiana benthamiana*. Collectively, these analyses establish a foundational framework for exploring the roles of *PsPEBP* genes in reproductive development.

## 2. Materials and Methods

### 2.1. Plant Materials and Sample Collection

Floral specimens of *Prunus salicina* ‘Fengtangli’ were procured from healthy, five-year-old trees located at the Guiyang experimental orchard of the Guizhou Institute of Pomology Science (26° N, 106° E). The collection focused on healthy floral buds and flowers from the outer canopy. The sampling process involved three biological replicates, each consisting of 20 floral buds or flowers sourced from three distinct trees. Within each replicate, samples were pooled based on developmental stage (F1–F5). Immediately following collection, all samples were frozen in liquid nitrogen and subsequently stored at −80 °C until RNA extraction was performed.

### 2.2. Genome-Wide Identification of PsPEBP Family Members

The chromosome-level genome assembly of *Prunus salicina*, as reported by Liu et al. [31], along with its predicted protein sequences and GFF3 annotations, served as the reference dataset for this study. The hidden Markov model (HMM) profile for the PEBP domain (PF01161) was sourced from Pfam [34] and employed to query the predicted proteome using HMMER version 3.3 [35], with an E-value threshold set at 1 × 10^−5^. Concurrently, PEBP protein sequences from *Arabidopsis thaliana*, obtained from TAIR [36], were utilized as query sequences in BLASTp 2.14.0+ searches against the same proteome, maintaining an E-value threshold of ≤1 × 10^−5^ [37]. The candidates identified through both methodologies were consolidated, and redundant entries were eliminated. Protein domain assignments were further evaluated using Pfam [34], SMART [38], and the NCBI Conserved Domain Database (CDD) [39]. Ultimately, seven non-redundant candidate genes were identified and designated as *PsPEBP1*–*PsPEBP7*. *PsPEBP4* was retained as a provisional candidate pending confirmation of the completeness of its unusually short gene model.

### 2.3. Physicochemical Property Analysis

Theoretical isoelectric point (pI), molecular weight (MW), instability index, aliphatic index, and grand average of hydropathicity (GRAVY) of each PsPEBP protein were calculated using ExPASy ProtParam [40]. Subcellular localization was predicted using WoLF PSORT [41].

### 2.4. Chromosomal Localization and Phylogenetic Analysis

Chromosomal positions of *PsPEBP* genes were retrieved from the GFF3 file and visualized using TBtools v2.441 [42]. For phylogenetic reconstruction, PEBP protein sequences from *P. salicina*, *Arabidopsis thaliana*, *Prunus persica*, *Prunus mume*, *Prunus armeniaca*, *Prunus avium*, *Prunus dulcis*, *Malus domestica*, *Pyrus bretschneideri*, *Fragaria vesca*, and *Rosa chinensis* were aligned with MAFFT and used to construct a neighbor-joining (NJ) tree in MEGA-X [43,44] with 1000 bootstrap replicates. The resulting tree was visualized alongside copy-number comparisons of the PEBP family in each Rosaceae species.

### 2.5. Gene Structure and Conserved Motif Analysis

Exon–intron structures of *PsPEBP* genes were visualized using GSDS 2.0 [45] based on the corresponding GFF3 annotations and coding sequences. Conserved motifs in PsPEBP proteins were identified using MEME [46], with the maximum number of motifs set to 10 and all other parameters retained at their default settings. Three-dimensional protein structures were predicted by homology modeling using the SWISS-MODEL server [47].

### 2.6. Synteny and Selection Pressure Analysis

Intraspecific collinearity within *P. salicina* and interspecific collinearity between *P. salicina* and *P. persica* or *Arabidopsis thaliana* were analyzed using MCScanX [48] and visualized using TBtools [42]. Nonsynonymous (*Ka*) and synonymous (*Ks*) substitution rates were calculated for each syntenic gene pair using the *Ka*/*Ks* calculation function in TBtools [42]. The ratio ω = *Ka*/*Ks* was used to assess selective constraints. Ratios below, close to, or above 1 were interpreted as being consistent with purifying selection, neutral evolution, or positive selection, respectively.

### 2.7. Cis-Acting Element Analysis

The 2000 base pair genomic regions upstream of the transcription start site for each *PsPEBP* gene were extracted and subjected to analysis using PlantCARE [49] to identify potential cis-acting regulatory elements. The predicted elements were categorized into four functional groups: light-responsive, hormone-responsive, development-related, and stress-responsive. Subsequently, their distributions were visualized.

### 2.8. Transcriptomic and qRT-PCR Analyses

Public RNA-seq data for *Prunus salicina* ‘Furongli’ fruits at 105, 115, 125, and 135 days after flowering (DAF) were retrieved from the NCBI Sequence Read Archive under BioProject PRJNA323640 [33]. Expression values for *PsPEBP* genes, expressed as fragments per kilobase of transcript per million mapped reads (FPKM), were extracted and visualized as a heatmap using the R package pheatmap [50].

For qRT-PCR analysis, three biological replicates were collected at each developmental stage (F1–F5) from ‘Fengtangli’, with each replicate consisting of a pool of 20 floral buds or flowers at the same developmental stage. Total RNA was extracted from each pooled sample using the MolPure^®^ TRIeasy™ Plus Total RNA Kit (Yeasen, Shanghai, China), and first-strand cDNA was synthesized using Hifair^®^ III 1st Strand cDNA Synthesis SuperMix (Yeasen). Gene-specific primers (Table S1) were designed using Primer3 [51] and synthesized by Sangon Biotech (Shanghai, China). PsActin was used as the reference gene based on its previous use in ‘Fengtangli’ [30], although its expression stability across F1–F5 was not independently assessed.

qRT-PCR was performed on a CFX96 Real-Time PCR Detection System (Bio-Rad Laboratories, Inc., Hercules, CA, USA) using Hieff^®^ qPCR SYBR Green Master Mix (Yeasen Biotechnology Co., Ltd., Shanghai, China) in 20 µL reactions, with three technical replicates per biological replicate. The thermal program consisted of 95 °C for 5 min, followed by 40 cycles of 95 °C for 10 s and 60 °C for 30 s. Relative expression levels were calculated using the 2^−ΔΔCT^ method [52], with F1 as the calibrator for each target gene. Statistical analyses and plotting were performed in GraphPad Prism 8.0. For each gene, differences among developmental stages were assessed using one-way ANOVA followed by Tukey’s multiple comparisons test, with significance set at *p* < 0.05.

### 2.9. Subcellular Localization of PsPEBP2

The coding sequence of PsPEBP2, excluding the stop codon, was amplified using primers C302-F (5′-ttggagaggacagggtacccATGGCTTCCAGTGAAGAGTTC-3′) and C302-R (5′-gtcgactctagaggatccccCGAAAATGGCAATCAAGACCGC-3′), and subsequently inserted into the pCAMBIA2300-35S-EGFP vector to create the 35S::*PsPEBP2*–EGFP fusion construct. An empty vector expressing EGFP alone was utilized as a control. Both constructs were introduced into Agrobacterium tumefaciens strain GV3101, and the resulting bacterial suspensions were infiltrated into the abaxial surfaces of leaves from four-week-old *Nicotiana benthamiana* plants. After a 48 h incubation period in darkness, EGFP fluorescence was assessed using a laser scanning confocal microscope with excitation at 488 nm and emission detection between 500 and 550 nm. Chloroplast autofluorescence was recorded with excitation at 640 nm. Two biological replicates, corresponding to independent infiltrations, were analyzed, with at least three fields of view recorded per replicate.

## 3. Results

### 3.1. Identification and Physicochemical Properties of PsPEBPs

Seven candidate *PEBP* genes, labeled as *PsPEBP1* through *PsPEBP7*, were identified within the *P. salicina* genome (refer to Table 1). The proteins predicted from these genes comprised between 100 and 174 amino acids, exhibiting molecular weights ranging from 11.50 to 19.64 kDa and theoretical isoelectric points (pI) spanning 5.75 to 9.52. Notably, PsPEBP2 and PsPEBP4 demonstrated acidic theoretical pI values, whereas the remaining proteins exhibited basic pI values. According to the instability index, PsPEBP2, PsPEBP5, and PsPEBP6 were classified as predicted stable proteins, while the other four were deemed predicted unstable proteins. All seven proteins displayed negative GRAVY values (ranging from −0.438 to −0.046), suggesting a predominantly hydrophilic nature. Furthermore, WoLF PSORT analysis predicted chloroplast localization for PsPEBP3 and cytoplasmic localization for the other six proteins.

### 3.2. Chromosomal Localization and Phylogenetic Classification

The seven *PsPEBP* genes were mapped to five distinct chromosomes: LG01, LG02, LG05, LG07, and LG08 (Figure 1). Chromosome LG01 harbored *PsPEBP1*, *PsPEBP2*, and *PsPEBP3*, while each of the remaining chromosomes contained a single gene. Through comparative phylogenetic analysis, *PsPEBP1*, *PsPEBP5*, and *PsPEBP7* were categorized within the TFL1-like group, *PsPEBP3* was classified under the FT-like group, and *PsPEBP6* was identified as part of the MFT-like group. *PsPEBP2* and *PsPEBP4* were associated with an AT5G01300-related group (Figure S1), although the completeness of the *PsPEBP4* gene model, which is notably short, remains unresolved. Accordingly, phylogenetic placement and any downstream structural comparison involving *PsPEBP4* should be treated as provisional.

The assembled sequence set for this comparative analysis comprised ten species from the Rosaceae family, along with *Arabidopsis thaliana* (Figure 2). Within this dataset, the total number of candidate sequences identified were as follows: six in *Prunus persica*, *Prunus armeniaca*, and *Prunus mume*; seven in *Prunus salicina* and *Prunus avium*; eight in *Prunus dulcis*, *Fragaria vesca*, and *Rosa chinensis*; and eleven in *Malus domestica* and *Pyrus bretschneideri*.

### 3.3. Conserved Motifs, Gene Structures, and Predicted Protein Structures

Ten conserved motifs were identified among the predicted PsPEBP proteins (Figure 3A). The proteins PsPEBP1, PsPEBP3, PsPEBP5, PsPEBP6, and PsPEBP7 exhibited all ten motifs in an identical sequence. In contrast, PsPEBP2 and PsPEBP4 displayed motifs 1 through 9 in the same order, but motif 10 was absent. Notably, the motifs in PsPEBP4 were confined to a shorter segment of the predicted protein sequence. Gene structure analysis indicated that PsPEBP1, PsPEBP3, PsPEBP5, PsPEBP6, and PsPEBP7 each comprised four exons and three introns. Conversely, PsPEBP2 was characterized by two exons and one intron, whereas PsPEBP4 contained three exons and two introns (Figure 3B). The predicted three-dimensional models of PsPEBP1, PsPEBP2, PsPEBP3, PsPEBP5, PsPEBP6, and PsPEBP7 demonstrated a broadly similar architecture, rich in β-sheets, accompanied by α-helices and connecting loops. The model for PsPEBP4, however, exhibited fewer β-strands and a less extensive β-sheet configuration, which is consistent with its shorter predicted sequence (Figure S2). These predicted features of PsPEBP4 are based on the current gene model, whose completeness remains unverified.

### 3.4. Selection Pressure and Synteny

All analyzed *PsPEBP*-associated orthologous and paralogous gene pairs had lower nonsynonymous substitution rates (*Ka*) than synonymous substitution rates (*Ks*) (Figure 4A). Their *Ka*/*Ks* ratios ranged from approximately 0.03 to 0.35, consistent with purifying selection (Figure 4B). The orthologous pair involving PsPEBP3 exhibited the lowest estimated ratio, approximately 0.03. The *PsPEBP1*–*PsPEBP7* paralogous pair had a Ks value of 0.96, compared with a broad density peak near 1.5 in the genome-wide Ks distribution of syntenic gene pairs (Figure S3).

Intraspecific synteny analysis revealed a collinear relationship between *PsPEBP1* and *PsPEBP7*, located on linkage groups LG01 and LG08, respectively (Figure 5A). This paralogous pair exhibited an estimated *Ka*/*Ks* ratio of approximately 0.16 (Figure 4B). Interspecific synteny analysis demonstrated numerous collinear blocks between *P. salicina* and both *P. persica* and *Arabidopsis thaliana* (Figure 5B). Syntenic counterparts in *P. persica* were identified for six *PsPEBP* genes; however, no corresponding syntenic anchor was detected for *PsPEBP4*. Additionally, five *PsPEBP* genes exhibited syntenic links with loci in *A. thaliana* (Figure 5B).

### 3.5. Cis-Acting Regulatory Elements

Eighteen types of putative cis-regulatory elements were identified in the 2000-bp upstream regions of the seven *PsPEBP* genes using PlantCARE (Figure 6). These included TATA-box and CAAT-box, together with motifs annotated as associated with light, hormones, stress, and developmental processes. TATA-box and CAAT-box were detected in all seven upstream regions. Among the light-, hormone-, stress-, and development-associated groups, light-associated motifs had the highest combined count. The GATA-motif was detected in all seven upstream regions, with 9–25 predicted occurrences and the highest count in *PsPEBP3*. Motifs with hormone-associated annotations included ABRE, AuxRE, TGA-element, P-box, GARE-motif, CGTCA-motif, and TGACG-motif. CGTCA-motif and TGACG-motif each showed their highest count in the upstream region of *PsPEBP7*, with four occurrences. Motifs with stress-associated annotations included MBS, LTR, ARE, and W-box; LTR was detected once in *PsPEBP6* and was not detected in the other analyzed regions. CAT-box, annotated as development-associated, was identified in five upstream regions, with the highest counts in *PsPEBP1* and *PsPEBP3*, each with four occurrences. These descriptive annotation groups do not imply mutually exclusive biological functions.

### 3.6. Expression Profiles of PsPEBP Genes During Fruit and Floral Development

The analysis of public fruit transcriptome data from ‘Furongli’ identified distinct temporal expression patterns among the four *PsPEBP* genes, as illustrated in Figure S4. Notably, *PsPEBP2*, *PsPEBP3*, and *PsPEBP5* exhibited their highest expression levels at 115 days after flowering (DAF), whereas *PsPEBP4* reached its peak expression at 125 DAF. Furthermore, the expression profiles of all seven *PsPEBP* genes in ‘Fengtangli’ were assessed using qRT-PCR across five consecutive stages of floral bud development and flower opening (F1–F5; Figure 7H). *PsPEBP1*, *PsPEBP2*, and *PsPEBP7* demonstrated their highest relative expression levels at stage F1, followed by a significant decline in subsequent stages (Figure 7A,B,G). *PsPEBP3* and *PsPEBP6* reached their peak expression at stage F2, with levels approximately three and ten times higher than those at F1, respectively. The expression of *PsPEBP3* subsequently decreased through stage F4 before increasing again at F5, whereas *PsPEBP6* experienced a sharp decline at F3 and remained low through F5 (Figure 7C,F). *PsPEBP4* expression decreased at F2 and then progressively increased to its highest level at F5 (Figure 7D). *PsPEBP5* displayed similar expression levels at F1 and F2, peaked at F3, and maintained levels above those at F1 during stages F4–F5 (Figure 7E).

### 3.7. PsPEBP2 Exhibits Cytoplasmic and Nuclear Localization

The 35S::*PsPEBP2*–EGFP fusion construct was transiently expressed in *Nicotiana benthamiana* leaves, with 35S::EGFP serving as the control (Figure 8A). Subcellular localization analysis indicated that PsPEBP2 was localized to both the cytoplasm and nucleus in leaf epidermal cells (Figure 8B). No clear overlap was observed between *PsPEBP2*–EGFP fluorescence and chloroplast autofluorescence. These results support a cytoplasmic and nuclear distribution of PsPEBP2 under the transient expression conditions used.

## 4. Discussion

Seven candidate *PsPEBP* genes were identified in Chinese plum and categorized into four distinct groups: TFL1-like (*PsPEBP1*, *PsPEBP5*, and *PsPEBP7*), FT-like (*PsPEBP3*), MFT-like (*PsPEBP6*), and AT5G01300-related (*PsPEBP2* and *PsPEBP4*). The recognition of the fourth group aligns with the identification of *AT5G01300* as *STEPMOTHER OF FT AND TFL1* (*SMFT*), which represents a unique plant PEBP lineage [10]. This expanded classification offers a more suitable framework for interpreting the seven candidates compared to the traditional three-group scheme. A previous comparative study identified five to six *PEBP* genes in the analyzed *Prunus* genomes using a three-group classification and complete-domain screening [26]. Therefore, variations in family definitions and the completeness of annotations must be considered before attributing differences in gene number to lineage-specific expansion or contraction.

The conserved motif organization, exon–intron structures, and predicted protein folds suggest significant sequence and structural conservation among several PsPEBP members (Figure 3 and Figure S2). Notably, PsPEBP2 and PsPEBP4 lack a detectable motif 10, setting their motif profiles apart from the other five proteins. While these differences offer valuable features for group comparison, their functional significance remains unresolved. Experimental studies in *Arabidopsis* have demonstrated that specific amino acid residues and a divergent external loop contribute to the contrasting activities of FT and TFL1, despite their similar overall folds [17,18]. Therefore, structural resemblance alone is insufficient to determine flowering-promoting or flowering-repressing activity. The shorter sequence of PsPEBP4 and its less extensive predicted β-sheet arrangement necessitate particular caution; comprehensive transcript and gene-model verification are required before these features can be interpreted as indicative of biological structural divergence.

The observed *Ka*/*Ks* ratios, ranging from approximately 0.03 to 0.35, indicate purifying selection acting on the coding sequences of the orthologous and paralogous pairs analyzed (Figure 4). Notably, the pair involving PsPEBP3 exhibited the lowest estimate; however, it is important to recognize that pairwise substitution-rate estimates can be influenced by the assumptions underlying evolutionary models [53]. This numerical ranking does not definitively indicate a uniquely strong selective constraint or a specific developmental function. Syntenic relationships with peach and *Arabidopsis* were identified for six and five *PsPEBP* genes, respectively, suggesting the conservation of genomic relationships among several family members (Figure 5). Within the Chinese plum, the PsPEBP1–PsPEBP7 pair maintained collinearity and exhibited a *Ka*/*Ks* ratio of approximately 0.16. The *Ks* value of 0.96, when considered in the context of the genome-wide distribution, provides insight into sequence divergence but does not, in isolation, pinpoint the duplication event responsible for this pair (Figure S3).

The identification of *PsPEBP3* as a priority candidate for functional investigation during floral development is supported by its FT-like assignment and the peak expression of F2. Functional studies in woody fruit crops provide relevant precedents: overexpression of *MdFT1* induces early flowering in apple, and the expression of poplar *FT1* modifies flowering and other developmental traits in European plum (*P. domestica*) [27,29]. These findings suggest that investigating the activity of *PsPEBP3* in Chinese plum is warranted. However, it is important to note that the F2 peak was observed during the floral bud swelling stage, after the formation of floral buds. Consequently, the current study focuses on bud development and flower opening rather than the initial floral transition. Furthermore, the antagonistic activities of the FT homologs *BvFT1* and *BvFT2* in sugar beet illustrate that membership in the FT-like group does not necessarily predict the promotion of flowering [23].

The differential expression profiles of the remaining members indicate variations in their temporal regulation (Figure 7). Within the TFL1-like gene group, *PsPEBP1* and *PsPEBP7* exhibited peak expression at stage F1, whereas *PsPEBP5* reached its peak at stage F3. The role of TFL1 in maintaining meristem identity in *Arabidopsis*, along with the shortened juvenile phase observed following *MdTFL1* suppression in apple, provides a framework for investigating similar functions in these candidates [4,28]. However, their expression profiles alone do not confirm antagonistic interactions with *PsPEBP3*. The peak expression of the AT5G01300-related *PsPEBP2* at stage F1, along with the gradual increase in *PsPEBP4* expression post-F2, further differentiates these two members of the group. Additionally, the approximately tenfold increase in expression of the MFT-like *PsPEBP6* at stage F2 warrants further investigation. In *Arabidopsis*, MFT is involved in regulating seed germination through ABA-associated signaling pathways, while SMFT influences seed dormancy and germination characteristics without affecting flowering time under the tested conditions [9,10]. These observations underscore the importance of exploring broader developmental roles for the corresponding plum candidates. Since F1 is morphologically characterized as an early floral bud stage, attributing the early expression peaks to dormancy regulation is not feasible without an independent evaluation of the dormancy status. Due to differences in cultivar, organ, and measurement method between the fruit transcriptomic and floral qRT-PCR datasets, these datasets were analyzed independently and were not employed for direct quantitative comparison or reciprocal validation.

The upstream regions contained 18 types of putative cis-regulatory elements, including TATA-box and CAAT-box, together with motifs annotated as associated with light, hormones, stress, and developmental processes (Figure 6). These annotation groups are descriptive and do not imply mutually exclusive biological functions. Among the light-, hormone-, stress-, and development-associated groups, light-associated motifs had the highest combined count, with the GATA-motif present in all seven upstream regions. Notably, there were 25 occurrences of the GATA-motif in *PsPEBP3*, four occurrences each of the CGTCA- and TGACG-motifs in *PsPEBP7*, and LTR was detected only in the analyzed upstream region of *PsPEBP6*, providing candidate motifs for further regulatory investigation. While PlantCARE annotations identify putative sequence elements [49], their presence alone does not confirm transcription-factor binding or responsiveness to the associated signals. Similarly, the detection of the CAT-box does not establish meristem-specific expression. To ascertain which predicted elements contribute to transcriptional regulation, promoter-reporter assays combined with motif mutagenesis and controlled environmental or hormone treatments could be used to test their regulatory activity.

The contrasting expression profiles of *PsPEBP1*/*PsPEBP7* and *PsPEBP5*, and of *PsPEBP2* and *PsPEBP4*, suggest divergence in developmental regulation within the same phylogenetic groups. The *PsPEBP3* peak during bud swelling is also noteworthy, given the increased expression of *PmFT* during floral bud development and blooming in *P. mume* [26]. This parallel supports investigating *PsPEBP3* in floral bud development, although its effect on flowering remains unverified. Developmental variation in fruit expression further suggests that selected *PsPEBP* members may have functions beyond floral development. Subcellular localization analysis showed that PsPEBP2 localized to both the cytoplasm and the nucleus in *Nicotiana benthamiana* leaf epidermal cells (Figure 8). Nevertheless, it is important to note that this result was derived from a heterologous expression system, and the localization of PsPEBP2 within its native cellular environment in *P. salicina* has yet to be confirmed. This finding provides a starting point for investigating the cellular role of PsPEBP2, an AT5G01300/SMFT-related protein whose function in Chinese plum remains uncharacterized. Established examples of PEBP-associated signaling include FT-FD interactions in *Arabidopsis* and the Hd3a-14-3-3-OsFD1 florigen activation complex in rice [13,14]; however, analogous interactions have not been demonstrated for PsPEBP2. Further research is needed to elucidate its endogenous distribution, interaction partners, and compartment-specific activities.

## 5. Conclusions

Seven candidate *PsPEBP* genes were identified in Chinese plum and categorized into the TFL1-like, FT-like, MFT-like, and AT5G01300-related groups. Comparative analyses revealed both conserved features and variations in gene structure and motif composition, while the homologous pairs analyzed exhibited *Ka*/*Ks* ratios indicative of purifying selection. Expression profiling demonstrated distinct temporal patterns during fruit development, floral bud development, and flower opening, with the FT-like *PsPEBP3* emerging as a candidate for functional investigation. PsPEBP2 showed both cytoplasmic and nuclear localization in transiently transformed *N. benthamiana* leaf cells. These findings provide a foundation for further exploration of PEBP functions in the reproductive development of Chinese plum.

## Figures and Tables

**Figure 1:**
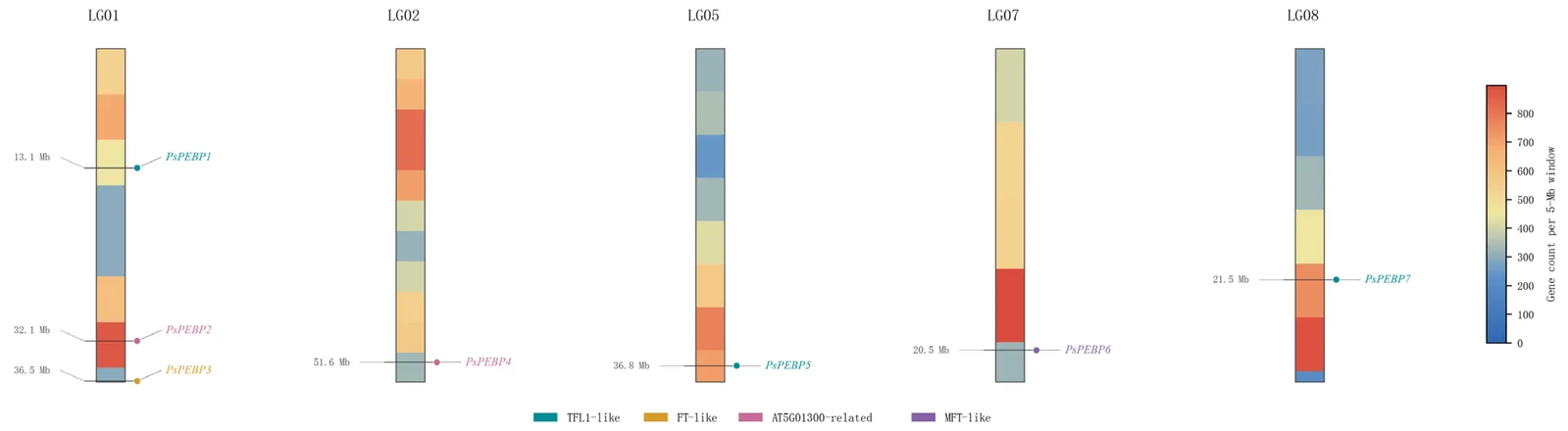
Chromosomal distribution of the seven *PsPEBP* genes in *Prunus salicina*.

**Figure 2:**
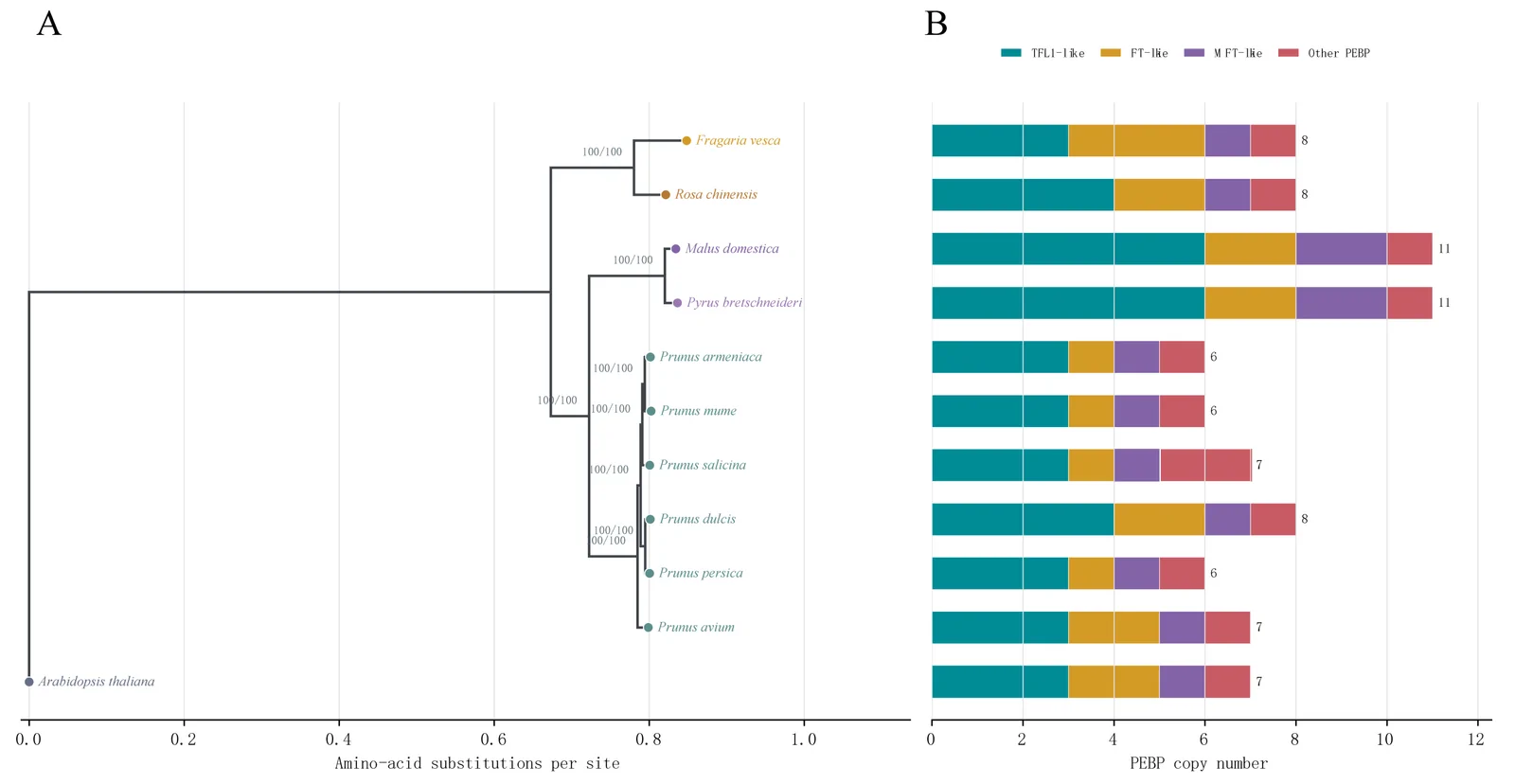
Species relationships and PEBP copy numbers in ten Rosaceae species and *Arabidopsis thaliana*. (**A**) Relationships among the analyzed species, with *A. thaliana* shown as the outgroup. (**B**) PEBP copy numbers in the corresponding species. Stacked bars show TFL1-like, FT-like, MFT-like, and other PEBP members, as indicated by the legend. Numbers at the ends of the bars indicate total copy numbers.

**Figure 3:**
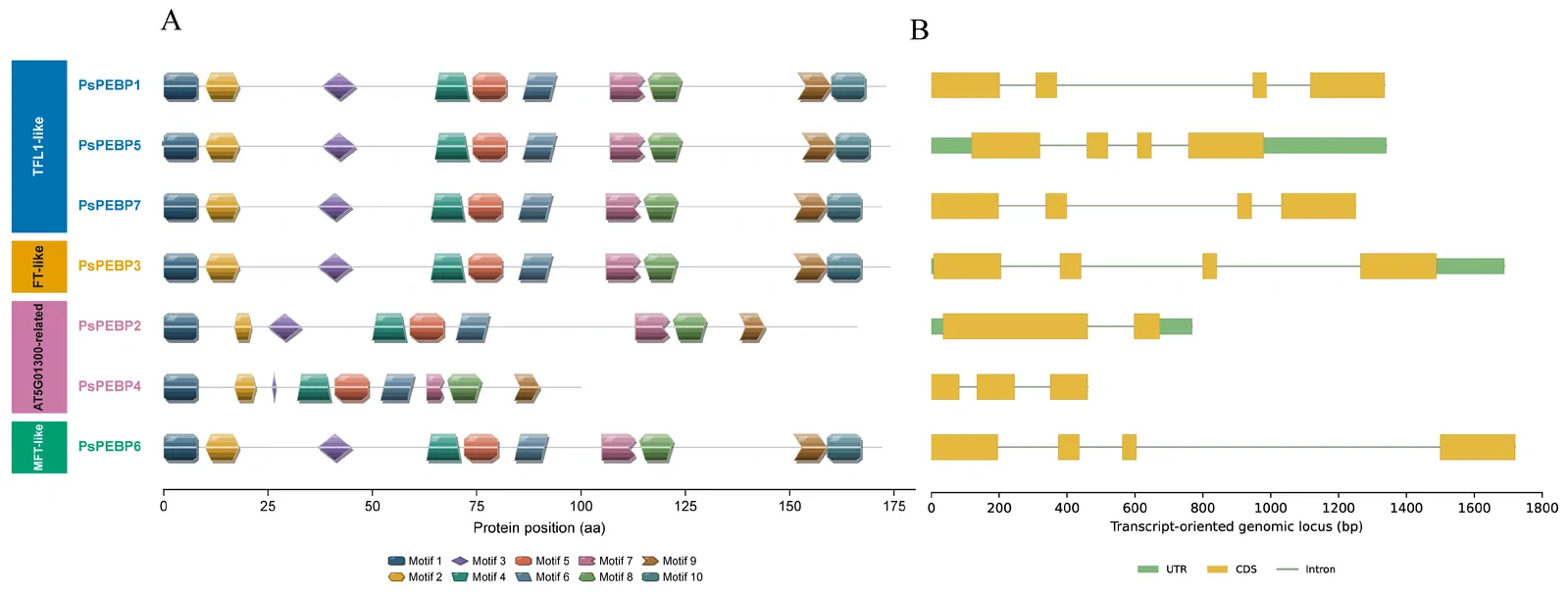
Conserved motif organization and gene structures of PsPEBP family members. (**A**) Distribution of ten conserved motifs in the seven PsPEBP proteins. Differently colored boxes represent motifs 1–10, with their positions and widths corresponding to their locations and lengths in the protein sequences. Gray lines represent regions outside the identified motifs. The horizontal scale indicates protein length in amino acids (aa). (**B**) Exon–intron organization of the corresponding PsPEBP genes. Yellow boxes indicate coding sequences (CDS), green boxes indicate untranslated regions (UTRs), and connecting lines indicate introns. Gene structures are displayed in the direction of transcription, with lengths indicated in base pairs (bp). Rows correspond between panels.

**Figure 4:**
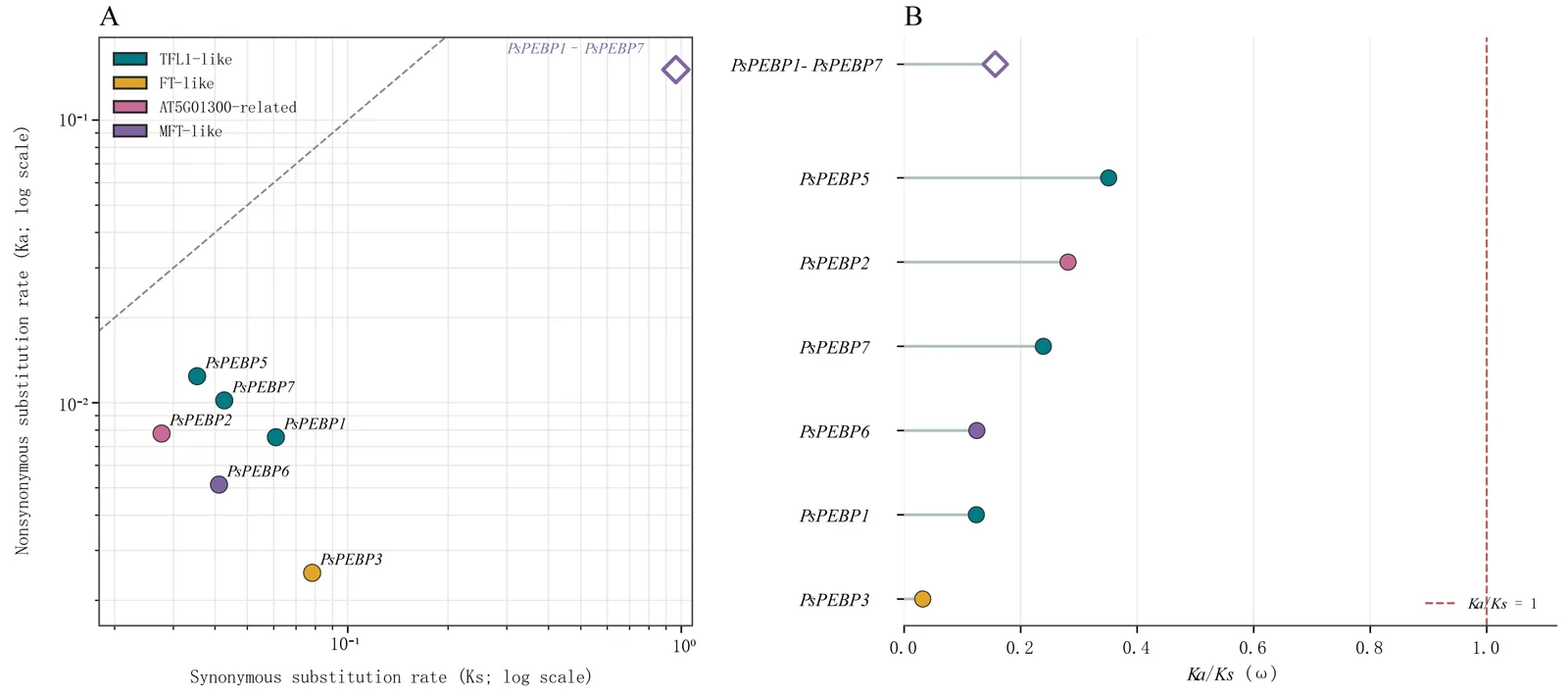
Selection pressure analysis of *PsPEBP*-associated orthologous and paralogous gene pairs. (**A**) Nonsynonymous substitution rates (*Ka*) plotted against synonymous substitution rates (*Ks*), with both axes displayed on logarithmic scales. The gray dashed diagonal indicates *Ka* = *Ks*. (**B**) Estimated Ka/Ks ratios for the analyzed gene pairs. The red dashed line indicates *Ka*/*Ks* = 1. Filled circles represent interspecific orthologous pairs labeled by their corresponding *P. salicina* gene, whereas the open diamond represents the intraspecific paralogous pair PsPEBP1–PsPEBP7. The colors of the filled circles distinguish the TFL1-like, FT-like, MFT-like, and AT5G01300-related groups, as indicated in the legend.

**Figure 5:**
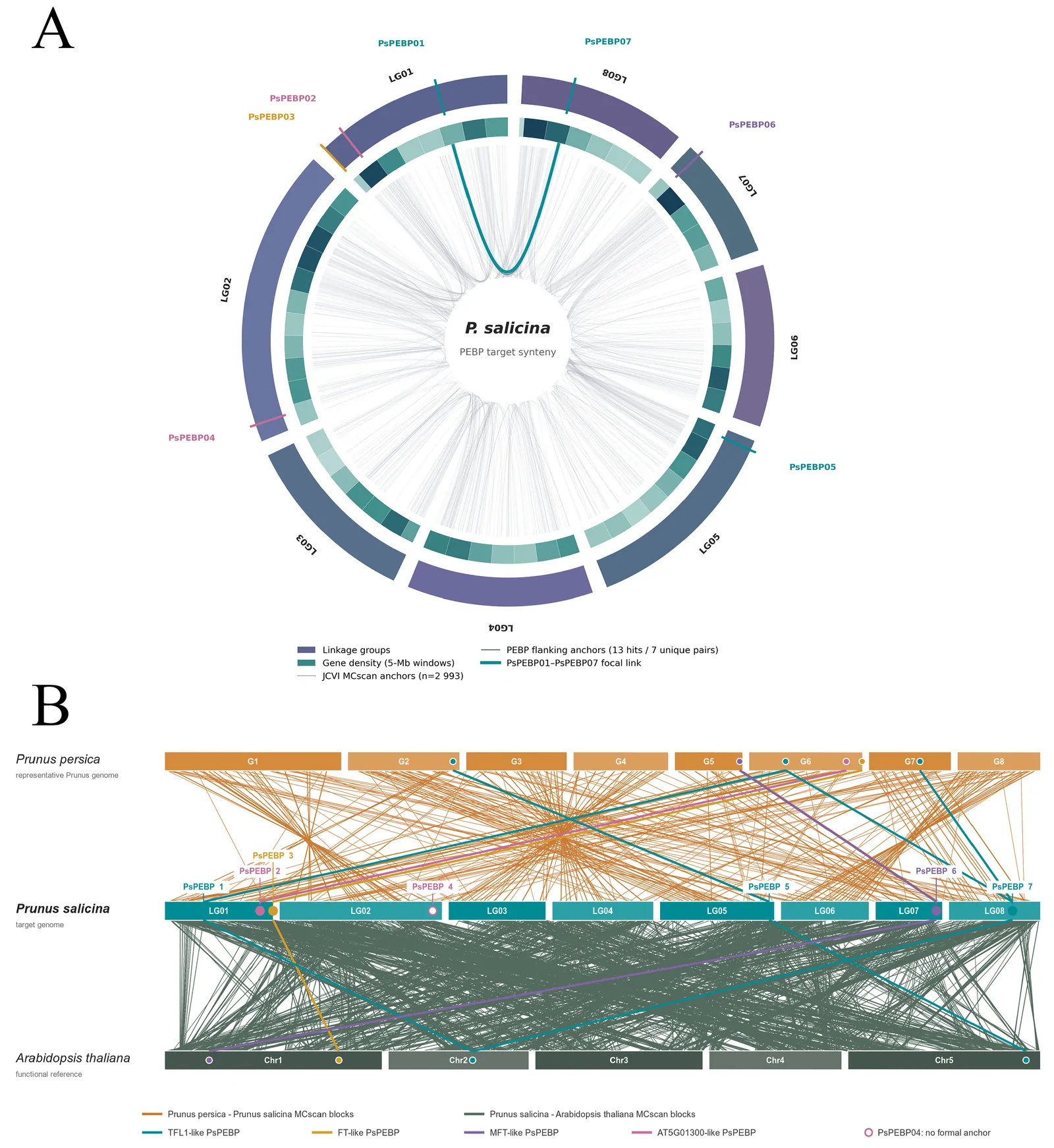
Intraspecific and interspecific synteny analyses of PsPEBP genes. (**A**) Intraspecific synteny in *Prunus salicina*. The outer ring represents the eight linkage groups, and the inner heatmap shows gene density in 5-Mb windows. Light gray lines represent genome-wide syntenic anchors, whereas darker gray lines indicate anchors flanking PsPEBP loci. The highlighted teal link connects the paralogous pair PsPEBP1–PsPEBP7. (**B**) Interspecific synteny between *P. salicina* and *P. persica* or *Arabidopsis thaliana*. Orange and dark green background lines represent genome-wide collinear blocks between *P. persica* and *P. salicina*, and between *P. salicina* and *A. thaliana*, respectively. Highlighted colored lines indicate PsPEBP-associated syntenic links. Gene labels and highlighted links are colored according to the TFL1-like, FT-like, MFT-like, and AT5G01300-related groups. The open pink circle marks PsPEBP4, for which no corresponding syntenic anchor was detected.

**Figure 6:**
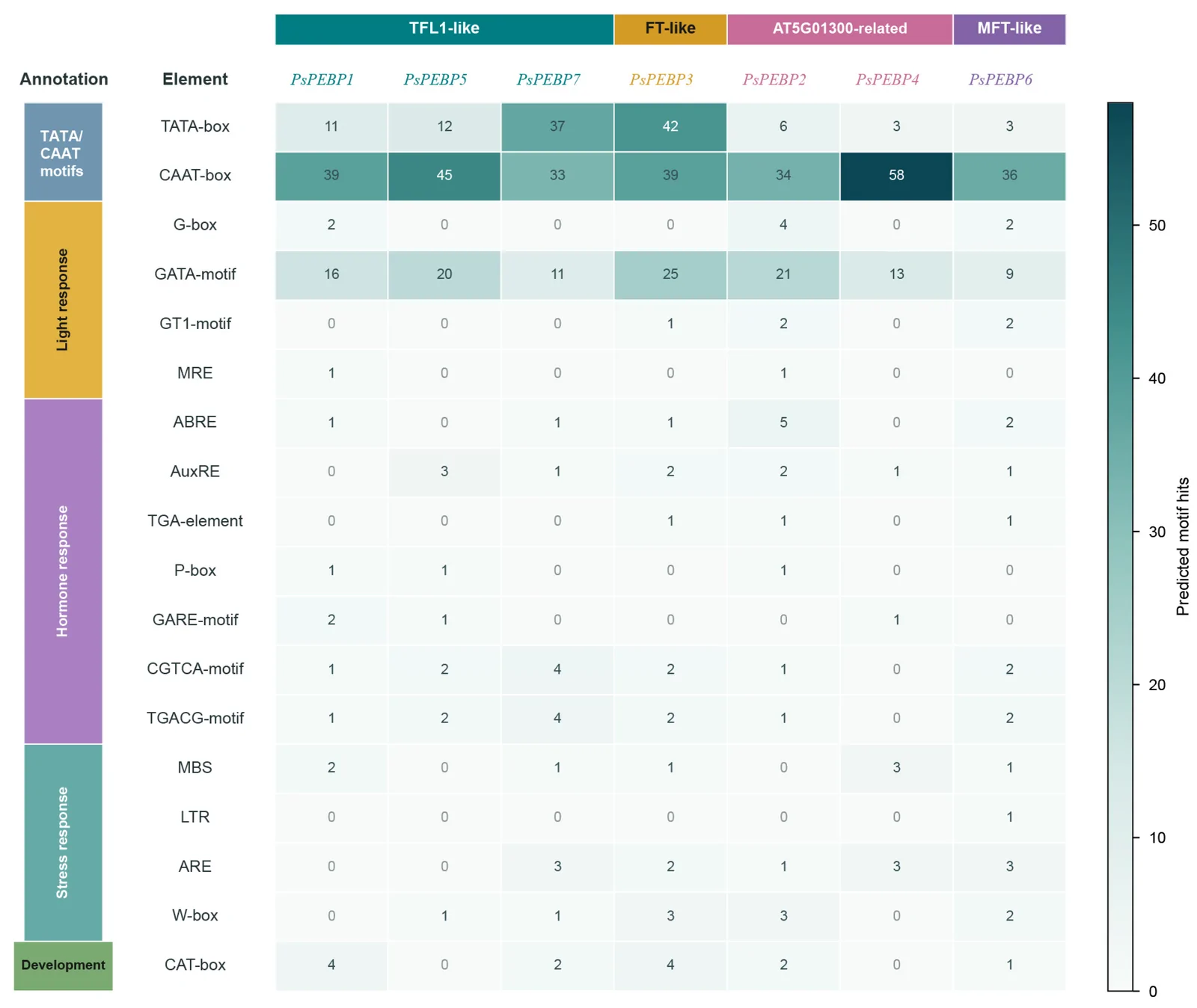
Distribution of predicted cis-regulatory motifs in the upstream regions of PsPEBP genes. The heatmap shows predicted motif counts in the 2000 bp upstream regions defined in Section 2.7. Columns represent the seven PsPEBP genes, and rows represent 18 putative cis-regulatory motif types. The colored bars on the left indicate descriptive annotation groups, with TATA-box and CAAT-box grouped under “TATA/CAAT motifs” and the remaining motifs organized according to their light-, hormone-, stress-, or development-associated annotations. These groups are used for visualization and do not imply mutually exclusive functions. The top bar indicates the TFL1-like, FT-like, AT5G01300-related, and MFT-like groups. Numbers within cells indicate predicted motif occurrences, with darker teal shading representing higher counts. Zero indicates that no matching motif was detected in the analyzed region. Motif occurrence alone does not demonstrate regulatory activity in *P. salicina*.

**Figure 7:**
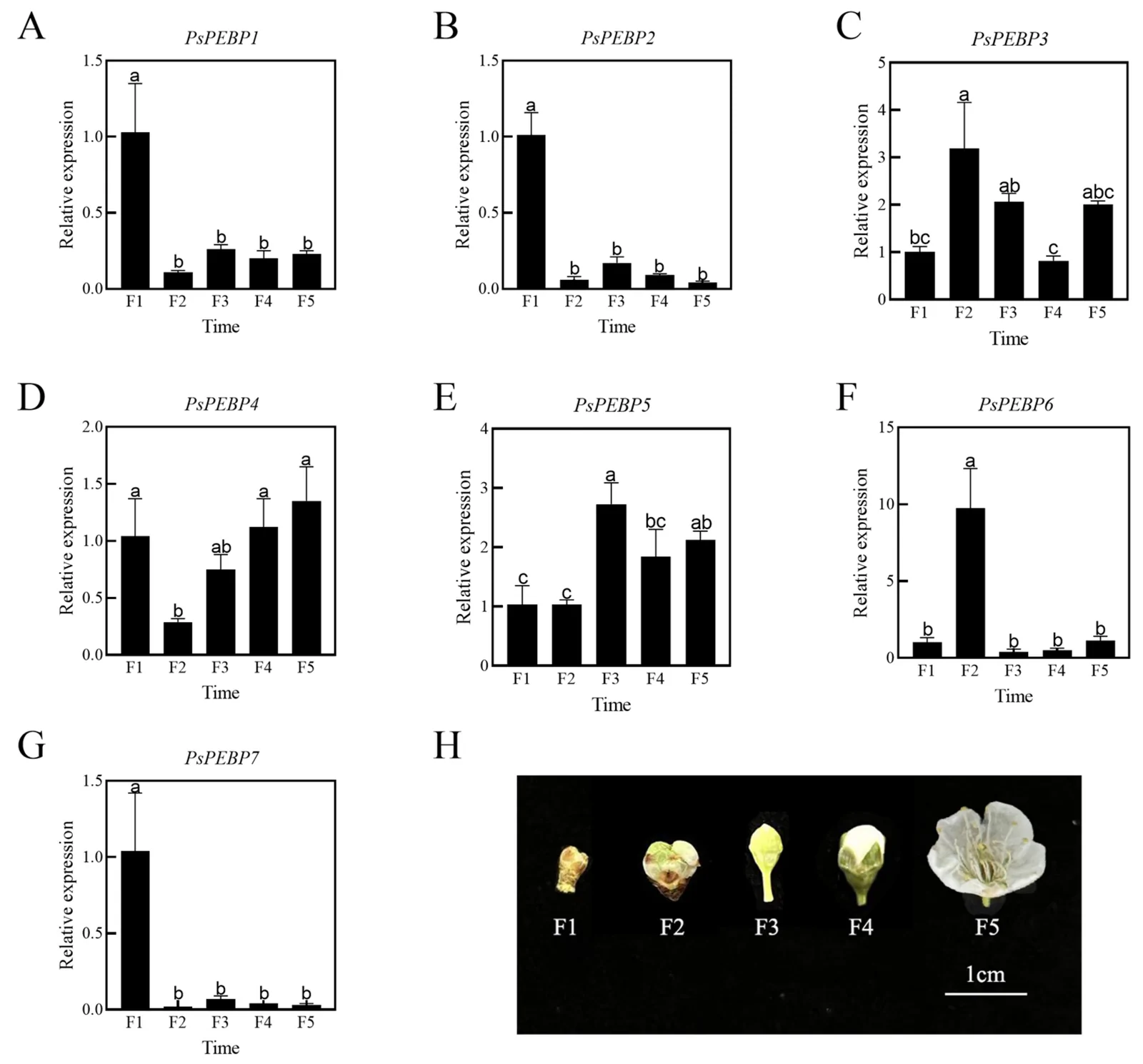
Relative expression profiles of *PsPEBP* genes during floral bud development and flower opening in ‘Fengtangli’. (**A**–**G**) Relative expression levels of *PsPEBP1*–*PsPEBP7*, respectively, determined by qRT-PCR. Expression levels were normalized to Actin, with F1 serving as the calibrator for each target gene. Bars represent mean values from three independent biological replicates. Within each gene, groups that do not share a lowercase letter differ significantly. (**H**) Representative images of the five developmental stages: F1, early floral bud; F2, swollen floral bud; F3, elongated, closed floral bud; F4, pre-anthesis with visible petals; and F5, open flower. Scale bar = 1 cm.

**Figure 8:**
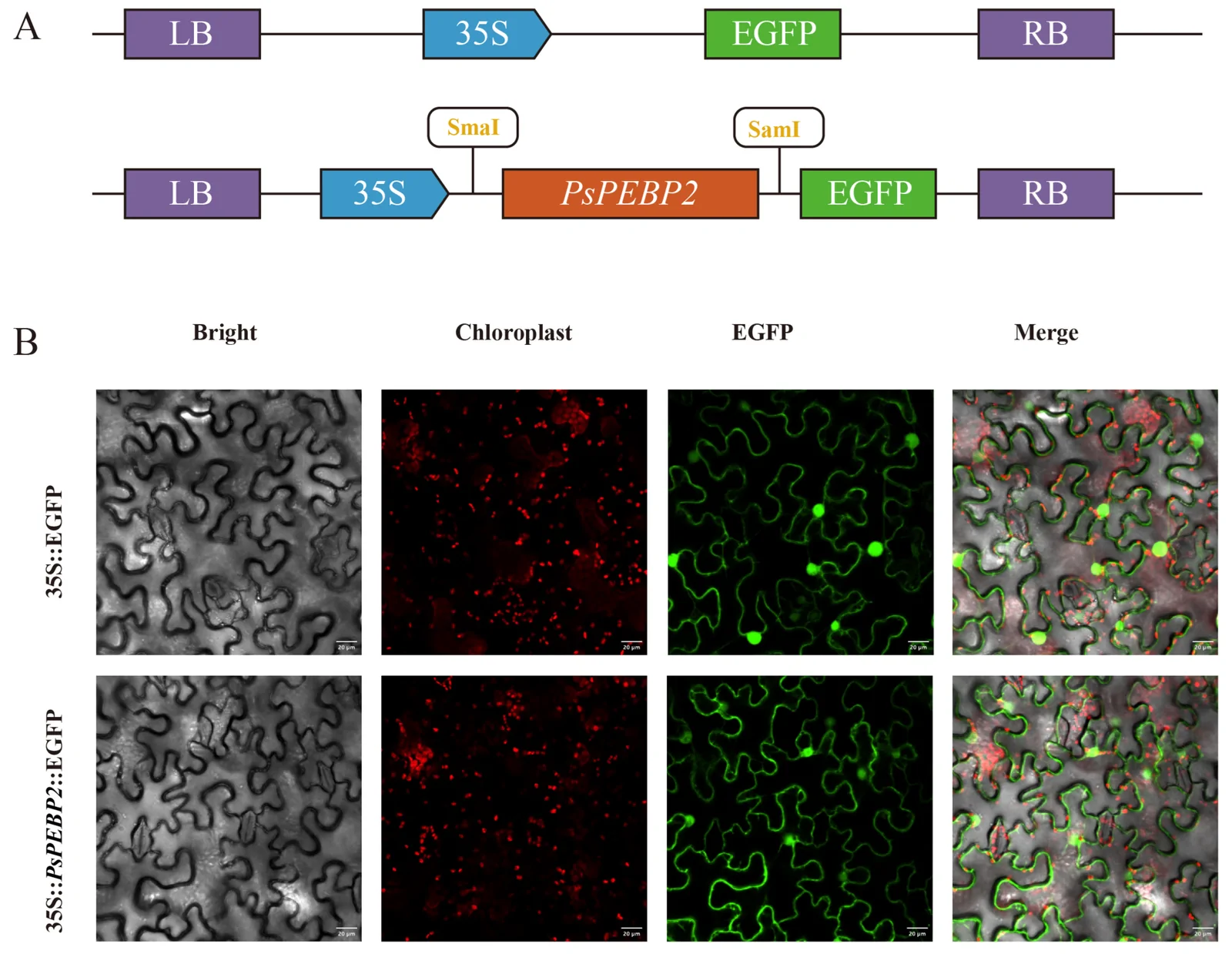
Subcellular localization analysis of PsPEBP2 in *Nicotiana benthamiana* leaf epidermal cells. (**A**) Schematic representation of the 35S::EGFP control and 35S::*PsPEBP2*–EGFP fusion constructs. EGFP was fused to the C terminus of *PsPEBP2*, and expression was driven by the cauliflower mosaic virus (CaMV) 35S promoter. LB and RB indicate the left and right T-DNA borders, respectively. (**B**) Representative confocal images of cells transiently expressing free EGFP (upper row) or *PsPEBP2*–EGFP (lower row). From left to right: bright-field images, chloroplast autofluorescence (red), EGFP fluorescence (green), and merged images. Scale bars = 20 μm.

**Table 1:** Characterization of PEBP family members in *Prunus salicina*.

Gene Name	Gene ID	Number of Amino Acids	Theoretical pI	Molecular Weight (Da)	Instability Index	Aliphatic Index	Average of Hydropathicity (GRAVY)	Predicted Subcellular Localization
*PsPEBP1*	PsSY0015626.1	173	9.52	19,495.38	40.99	79.31	−0.331	Cytoplasm
*PsPEBP2*	PsSY0026953.1	166	5.79	18,360.89	31.87	85.06	−0.438	Cytoplasm
*PsPEBP3*	PsSY0020170.1	174	7.72	19,577.22	42.11	79.94	−0.337	Chloroplast
*PsPEBP4*	PsSY0019977.1	100	5.75	11,498.33	42.22	101.40	−0.257	Cytoplasm
*PsPEBP5*	PsSY0012103.1	174	8.82	19,641.41	37.75	73.33	−0.401	Cytoplasm
*PsPEBP6*	PsSY0016889.1	172	8.79	18,877.83	39.39	84.30	−0.046	Cytoplasm
*PsPEBP7*	PsSY0008451.1	172	8.88	19,419.16	49.24	69.65	−0.342	Cytoplasm

## Data Availability

The public RNA-seq data for fruit development in *Prunus salicina* ‘Furongli’ analyzed in this study are available in the NCBI Sequence Read Archive under BioProject accession PRJNA323640.

## Supplementary Materials

The following supporting information can be downloaded at https://www.mdpi.com/article/10.3390/biology15191786/s1, Figure S1: Phylogenetic relationships of PEBP proteins from *Prunus salicina* and other selected plant species; Figure S2: Predicted three-dimensional structures of PsPEBP proteins; Figure S3: Genome-wide *Ks* distribution of syntenic gene pairs in *Prunus salicina*, highlighting the PsPEBP1–PsPEBP7 paralogous pair; Figure S4: Expression profiles of *PsPEBP* genes during fruit development in *Prunus salicina* ‘Furongli’; Table S1: Primers used in this study.

## References

[1] Samach A., Onouchi H., Gold S.E., Ditta G.S., Schwarz-Sommer Z., Yanofsky M.F., Coupland G. (2000). Distinct roles of CONSTANS target genes in reproductive development of *Arabidopsis*. Science.

[2] Searle I., He Y., Turck F., Vincent C., Fornara F., Kröber S., Amasino R.A., Coupland G. (2006). The transcription factor FLC confers a flowering response to vernalization by repressing meristem competence and systemic signaling in *Arabidopsis*. Genes Dev..

[3] Kobayashi Y., Kaya H., Goto K., Iwabuchi M., Araki T. (1999). A pair of related genes with antagonistic roles in mediating flowering signals. Science.

[4] Bradley D., Ratcliffe O., Vincent C., Carpenter R., Coen E. (1997). Inflorescence commitment and architecture in *Arabidopsis*. Science.

[5] Karlgren A., Gyllenstrand N., Källman T., Sundström J.F., Moore D., Lascoux M., Lagercrantz U. (2011). Evolution of the PEBP gene family in plants: Functional diversification in seed plant evolution. Plant Physiol..

[6] Yamaguchi A., Kobayashi Y., Goto K., Abe M., Araki T. (2005). TWIN SISTER OF FT (TSF) acts as a floral pathway integrator redundantly with FT. Plant Cell Physiol..

[7] Yoo S.J., Chung K.S., Jung S.H., Yoo S.Y., Lee J.S., Ahn J.H. (2010). BROTHER OF FT AND TFL1 (BFT) has TFL1-like activity and functions redundantly with TFL1 in inflorescence meristem development in *Arabidopsis*. Plant J..

[8] Huang N.C., Jane W.N., Chen J., Yu T.S. (2012). *Arabidopsis thaliana* CENTRORADIALIS homologue (ATC) acts systemically to inhibit floral initiation in *Arabidopsis*. Plant J..

[9] Xi W., Liu C., Hou X., Yu H. (2010). MOTHER OF FT AND TFL1 regulates seed germination through a negative feedback loop modulating ABA signaling in *Arabidopsis*. Plant Cell.

[10] Bellinazzo F., Nadal Bigas J., Hogers R.A.H., Kodde J., van der Wal F., Kokkinopoulou P., Duijts K.T.M., Angenent G.C., van Dijk A.D.J., van Velzen R. (2024). Evolutionary origin and functional investigation of the widely conserved plant PEBP gene *STEPMOTHER OF FT AND TFL1* (*SMFT*). Plant J..

[11] Corbesier L., Vincent C., Jang S., Fornara F., Fan Q., Searle I., Giakountis A., Farrona S., Gissot L., Turnbull C. (2007). FT protein movement contributes to long-distance signaling in floral induction of *Arabidopsis*. Science.

[12] Tamaki S., Matsuo S., Wong H.L., Yokoi S., Shimamoto K. (2007). Hd3a protein is a mobile flowering signal in rice. Science.

[13] Abe M., Kobayashi Y., Yamamoto S., Daimon Y., Yamaguchi A., Ikeda Y., Ichinoki H., Notaguchi M., Goto K., Araki T. (2005). FD, a bZIP protein mediating signals from the floral pathway integrator FT at the shoot apex. Science.

[14] Taoka K., Ohki I., Tsuji H., Furuita K., Hayashi K., Yanase T., Yamaguchi M., Nakashima C., Purwestri Y.A., Tamaki S. (2011). 14-3-3 proteins act as intracellular receptors for rice Hd3a florigen. Nature.

[15] Conti L., Bradley D. (2007). TERMINAL FLOWER1 is a mobile signal controlling *Arabidopsis* architecture. Plant Cell.

[16] Zhu Y., Klasfeld S., Jeong C.W., Jin R., Goto K., Yamaguchi N., Wagner D. (2020). TERMINAL FLOWER 1-FD complex target genes and competition with FLOWERING LOCUS T. Nat. Commun..

[17] Hanzawa Y., Money T., Bradley D. (2005). A single amino acid converts a repressor to an activator of flowering. Proc. Natl. Acad. Sci. USA.

[18] Ahn J.H., Miller D., Winter V.J., Banfield M.J., Lee J.H., Yoo S.Y., Henz S.R., Brady R.L., Weigel D. (2006). A divergent external loop confers antagonistic activity on floral regulators FT and TFL1. EMBO J..

[19] Pnueli L., Carmel-Goren L., Hareven D., Gutfinger T., Alvarez J., Ganal M., Zamir D., Lifschitz E. (1998). The *SELF-PRUNING* gene of tomato regulates vegetative to reproductive switching of sympodial meristems and is the ortholog of *CEN* and *TFL1*. Development.

[20] Lifschitz E., Eviatar T., Rozman A., Shalit A., Goldshmidt A., Amsellem Z., Alvarez J.P., Eshed Y. (2006). The tomato FT ortholog triggers systemic signals that regulate growth and flowering and substitute for diverse environmental stimuli. Proc. Natl. Acad. Sci. USA.

[21] Krieger U., Lippman Z.B., Zamir D. (2010). The flowering gene *SINGLE FLOWER TRUSS* drives heterosis for yield in tomato. Nat. Genet..

[22] Blackman B.K., Strasburg J.L., Raduski A.R., Michaels S.D., Rieseberg L.H. (2010). The role of recently derived FT paralogs in sunflower domestication. Curr. Biol..

[23] Pin P.A., Benlloch R., Bonnet D., Wremerth-Weich E., Kraft T., Gielen J.J.L., Nilsson O. (2010). An antagonistic pair of FT homologs mediates the control of flowering time in sugar beet. Science.

[24] Böhlenius H., Huang T., Charbonnel-Campaa L., Brunner A.M., Jansson S., Strauss S.H., Nilsson O. (2006). CO/FT regulatory module controls timing of flowering and seasonal growth cessation in trees. Science.

[25] Hsu C.-Y., Adams J.P., Kim H., No K., Ma C., Strauss S.H., Drnevich J., Vandervelde L., Ellis J.D., Rice B.M. (2011). FLOWERING LOCUS T duplication coordinates reproductive and vegetative growth in perennial poplar. Proc. Natl. Acad. Sci. USA.

[26] Zhang M., Li P., Yan X., Wang J., Cheng T., Zhang Q. (2021). Genome-wide characterization of PEBP family genes in nine Rosaceae tree species and their expression analysis in *P. mume*. BMC Ecol. Evol..

[27] Kotoda N., Hayashi H., Suzuki M., Igarashi M., Hatsuyama Y., Kidou S., Igasaki T., Nishiguchi M., Yano K., Shimizu T. (2010). Molecular Characterization of FLOWERING LOCUS T-Like Genes of Apple (*Malus × domestica* Borkh.). Plant Cell Physiol..

[28] Kotoda N., Iwanami H., Takahashi S., Abe K. (2006). Antisense Expression of *MdTFL1*, a TFL1-like Gene, Reduces the Juvenile Phase in Apple. J. Am. Soc. Hortic. Sci..

[29] Srinivasan C., Dardick C., Callahan A., Scorza R. (2012). Plum (*Prunus domestica*) Trees Transformed with Poplar *FT1* Result in Altered Architecture, Dormancy Requirement, and Continuous Flowering. PLoS ONE.

[30] Deng L., Wang T., Hu J., Yang X., Yao Y., Jin Z., Huang Z., Sun G., Xiong B., Liao L. (2022). Effects of Pollen Sources on Fruit Set and Fruit Characteristics of ‘Fengtangli’ Plum (*Prunus salicina* Lindl.) Based on Microscopic and Transcriptomic Analysis. Int. J. Mol. Sci..

[31] Liu C., Feng C., Peng W., Hao J., Wang J., Pan J., He Y. (2020). Chromosome-level draft genome of a diploid plum (*Prunus salicina*). GigaScience.

[32] Fang Z.-Z., Lin-Wang K., Dai H., Zhou D.-R., Jiang C.-C., Espley R.V., Deng C., Lin Y.-J., Pan S.-L., Ye X.-F. (2022). The genome of low-chill Chinese plum “Sanyueli” (*Prunus salicina* Lindl.) provides insights into the regulation of the chilling requirement of flower buds. Mol. Ecol. Resour..

[33] Fang Z.-Z., Zhou D.-R., Ye X.-F., Jiang C.-C., Pan S.-L. (2016). Identification of Candidate Anthocyanin-Related Genes by Transcriptomic Analysis of ‘Furongli’ Plum (*Prunus salicina* Lindl.) During Fruit Ripening Using RNA-Seq. Front. Plant Sci..

[34] Mistry J., Chuguransky S., Williams L., Qureshi M., Salazar G.A., Sonnhammer E.L.L., Tosatto S.C.E., Paladin L., Raj S., Richardson L.J. (2021). Pfam: The protein families database in 2021. Nucleic Acids Res..

[35] Eddy S.R. (2011). Accelerated Profile HMM Searches. PLoS Comput. Biol..

[36] Lamesch P., Berardini T.Z., Li D., Swarbreck D., Wilks C., Sasidharan R., Muller R., Dreher K., Alexander D.L., Garcia-Hernandez M. (2012). The Arabidopsis Information Resource (TAIR): Improved gene annotation and new tools. Nucleic Acids Res..

[37] Camacho C., Coulouris G., Avagyan V., Ma N., Papadopoulos J., Bealer K., Madden T.L. (2009). BLAST+: Architecture and applications. BMC Bioinform..

[38] Letunic I., Khedkar S., Bork P. (2021). SMART: Recent updates, new developments and status in 2020. Nucleic Acids Res..

[39] Wang J., Chitsaz F., Derbyshire M.K., Gonzales N.R., Gwadz M., Lu S., Marchler G.H., Song J.S., Thanki N., Yamashita R.A. (2023). The conserved domain database in 2023. Nucleic Acids Res..

[40] Gasteiger E., Hoogland C., Gattiker A., Duvaud S., Wilkins M.R., Appel R.D., Bairoch A., Walker J.M. (2005). Protein identification and analysis tools on the ExPASy server. The Proteomics Protocols Handbook.

[41] Horton P., Park K.J., Obayashi T., Fujita N., Harada H., Adams-Collier C.J., Nakai K. (2007). WoLF PSORT: Protein localization predictor. Nucleic Acids Res..

[42] Chen C., Chen H., Zhang Y., Thomas H.R., Frank M.H., He Y., Xia R. (2020). TBtools: An Integrative Toolkit Developed for Interactive Analyses of Big Biological Data. Mol. Plant.

[43] Katoh K., Standley D.M. (2013). MAFFT multiple sequence alignment software version 7: Improvements in performance and usability. Mol. Biol. Evol..

[44] Kumar S., Stecher G., Li M., Knyaz C., Tamura K. (2018). MEGA X: Molecular Evolutionary Genetics Analysis across Computing Platforms. Mol. Biol. Evol..

[45] Hu B., Jin J., Guo A.Y., Zhang H., Luo J., Gao G. (2015). GSDS 2.0: An upgraded gene feature visualization server. Bioinformatics.

[46] Bailey T.L., Boden M., Buske F.A., Frith M., Grant C.E., Clementi L., Ren J., Li W.W., Noble W.S. (2009). MEME SUITE: Tools for motif discovery and searching. Nucleic Acids Res..

[47] Waterhouse A., Bertoni M., Bienert S., Studer G., Tauriello G., Gumienny R., Heer F.T., de Beer T.A.P., Rempfer C., Bordoli L. (2018). SWISS-MODEL: Homology modelling of protein structures and complexes. Nucleic Acids Res..

[48] Wang Y., Tang H., DeBarry J.D., Tan X., Li J., Wang X., Lee T.-H., Jin H., Marler B., Guo H. (2012). MCScanX: A toolkit for detection and evolutionary analysis of gene synteny and collinearity. Nucleic Acids Res..

[49] Lescot M., Déhais P., Thijs G., Marchal K., Moreau Y., Van de Peer Y., Rouzé P., Rombauts S. (2002). PlantCARE, a database of plant cis-acting regulatory elements and a portal to tools for in silico analysis of promoter sequences. Nucleic Acids Res..

[50] Kolde R. pheatmap: Pretty Heatmaps. R package. https://CRAN.R-project.org/package=pheatmap.

[51] Untergasser A., Cutcutache I., Koressaar T., Ye J., Faircloth B.C., Remm M., Rozen S.G. (2012). Primer3—New capabilities and interfaces. Nucleic Acids Res..

[52] Livak K.J., Schmittgen T.D. (2001). Analysis of relative gene expression data using real-time quantitative PCR and the $2^{-\Delta \Delta C_{T}}$ method. Methods.

[53] Yang Z., Nielsen R. (2000). Estimating synonymous and nonsynonymous substitution rates under realistic evolutionary models. Mol. Biol. Evol..

